# Rapid and sensitive detection of early esophageal squamous cell carcinoma with fluorescence probe targeting dipeptidylpeptidase IV

**DOI:** 10.1038/srep26399

**Published:** 2016-06-01

**Authors:** Haruna Onoyama, Mako Kamiya, Yugo Kuriki, Toru Komatsu, Hiroyuki Abe, Yosuke Tsuji, Koichi Yagi, Yukinori Yamagata, Susumu Aikou, Masato Nishida, Kazuhiko Mori, Hiroharu Yamashita, Mitsuhiro Fujishiro, Sachiyo Nomura, Nobuyuki Shimizu, Masashi Fukayama, Kazuhiko Koike, Yasuteru Urano, Yasuyuki Seto

**Affiliations:** 1 Department of Gastrointestinal Surgery, Graduate School of Medicine, The University of Tokyo, 7-3-1 Hongo, Bunkyo-ku, Tokyo 113-8655, Japan; 2 Laboratory of Chemical Biology and Molecular Imaging, Graduate School of Medicine, The University of Tokyo, 7-3-1 Hongo, Bunkyo-ku, Tokyo 113-0033, Japan; 3 PRESTO, Japan and Science and Technology Agency, 4-1-8 Honcho, Kawaguchi, Saitama, 332-0012, Japan; 4 Laboratory of Chemistry and Biology, Graduate School of Pharmaceutical Sciences, The University of Tokyo, 7-3-1 Hongo, Bunkyo-ku, Tokyo 113-0033, Japan; 5 Department of Pathology, Graduate School of Medicine, The University of Tokyo, 7-3-1 Hongo, Bunkyo-ku, Tokyo 113-8655, Japan; 6 Department of Gastroenterology, Graduate School of Medicine, The University of Tokyo, 7-3-1 Hongo, Bunkyo-ku, Tokyo 113-8655, Japan; 7 Department of surgery, Dokkyo Medical University Koshigaya Hospital, 343-8555 2-1-50 Minami-Koshigaya, Koshigaya city, Saitama, Japan; 8 Mitsui Memorial Hospital, 1 Kanda-Izumi-cho, Chiyoda-ku, Tokyo 101-8643, Japan; 9 Sanno Hospital, International University of Health and Welfare, 8-10-16, Akasaka, Minato-ku, Tokyo 107-0052, Japan; 10 AMED-CREST, Japan Agency for Medical Research and Development, 1-7-1 Otemachi, Chiyoda-ku, Tokyo, 100-0004, Japan

## Abstract

Early detection of esophageal squamous cell carcinoma (ESCC) is an important prognosticator, but is difficult to achieve by conventional endoscopy. Conventional lugol chromoendoscopy and equipment-based image-enhanced endoscopy, such as narrow-band imaging (NBI), have various practical limitations. Since fluorescence-based visualization is considered a promising approach, we aimed to develop an activatable fluorescence probe to visualize ESCCs. First, based on the fact that various aminopeptidase activities are elevated in cancer, we screened freshly resected specimens from patients with a series of aminopeptidase-activatable fluorescence probes. The results indicated that dipeptidylpeptidase IV (DPP-IV) is specifically activated in ESCCs, and would be a suitable molecular target for detection of esophageal cancer. Therefore, we designed, synthesized and characterized a series of DPP-IV-activatable fluorescence probes. When the selected probe was topically sprayed onto endoscopic submucosal dissection (ESD) or surgical specimens, tumors were visualized within 5 min, and when the probe was sprayed on biopsy samples, the sensitivity, specificity and accuracy reached 96.9%, 85.7% and 90.5%. We believe that DPP-IV-targeted activatable fluorescence probes are practically translatable as convenient tools for clinical application to enable rapid and accurate diagnosis of early esophageal cancer during endoscopic or surgical procedures.

Esophageal cancer is the eighth most frequently diagnosed cancer worldwide and the sixth most common cause of cancer-related death; in particular, esophageal squamous cell carcinomas (ESCCs) are lifestyle-related, and risk factors include smoking and drinking alcohol^1^. Due to the asymptomatic nature of the disease in the early stages, esophageal cancer is often found at an advanced stage. Consequently, the overall prognosis of esophageal cancer is quite poor, with a five-year survival rate (5YSR) of around 14%^1^. However, when esophageal cancer is found at an early stage, there is a better chance of recovery; for instance, the 5YSR of esophageal cancer at stage 0 is more than 95%^1^. In addition, early detection enables minimally invasive curative treatment (e.g. endoscopic resection), which is much safer and less disruptive to patients^2^,^3^,^4^,^5^ compared to surgery, which involves a risk of postoperative complications, such as pneumonia, hoarseness from vocal cord nerve injury, leakage from the anastomosis and infection. Therefore, early detection of esophageal cancer is crucial for improvement of the survival rate and the health-related quality of life of patients.

In order to detect early-stage ESCCs in the absence of signs or symptoms, endoscopic screening of the esophagus is usually conducted with the aid of lugol chromoendoscopy or image-enhanced endoscopy, such as narrow-band imaging (NBI)^6^,^7^, since early-stage ESCCs are difficult to see in conventional endoscopy owing to the poor visual contrast between cancer and normal tissues under white light. However, instillation of lugol solution into the esophagus sometimes leads to complications (hypersensitivity to iodine, laryngitis, heartburn), and is not applicable repeatedly over a short period since it induces acute mucosal damage^8^,^9^,^10^. In contrast, NBI allows non-invasive observation, but it requires specialized expertise and training^11^,^12^, and the diagnostic yield requires further improvement.

On the other hand, fluorescence-guided detection has recently been investigated as an aid to optically guided endoscopy, because of its advantages of high sensitivity, low cost, and real-time capability^13^,^14^. We have already reported an activatable fluorescence imaging probe targeting γ-glutamyltranspeptidase (GGT), which is a membrane-bound protein known to be overexpressed in many types of cancer^15^. We recently confirmed the suitability of this probe, γ-glutamyl hydroxymethylrhodamine green (gGlu-HMRG), for detecting human breast cancer in resected tissue from patients^16^. However, we found that this probe is unsuitable for detecting ESCCs in clinical specimens, and we considered that a target other than GGT would be required for fluorescence-guided detection of esophageal cancers.

In the present work, we firstly employed a screening strategy to identify a suitable target aminopeptidase for visualizing esophageal cancer, using freshly resected specimens from patients and a series of aminopeptidase-activatable fluorescence probes. We identified dipeptidylpeptidase IV (DPP-IV) as a suitable target. We then designed, synthesized and characterized a series of HMRG-based fluorescent probes targeting DPP-IV. We confirmed that the selected probe could rapidly visualize tumors in freshly resected endoscopic submucosal dissection (ESD) specimens and surgical specimens with high sensitivity, specificity and accuracy.

## Results

### Screening of aminopeptidase activities of esophageal squamous cell carcinoma in human biopsy samples

**Figure 1:**
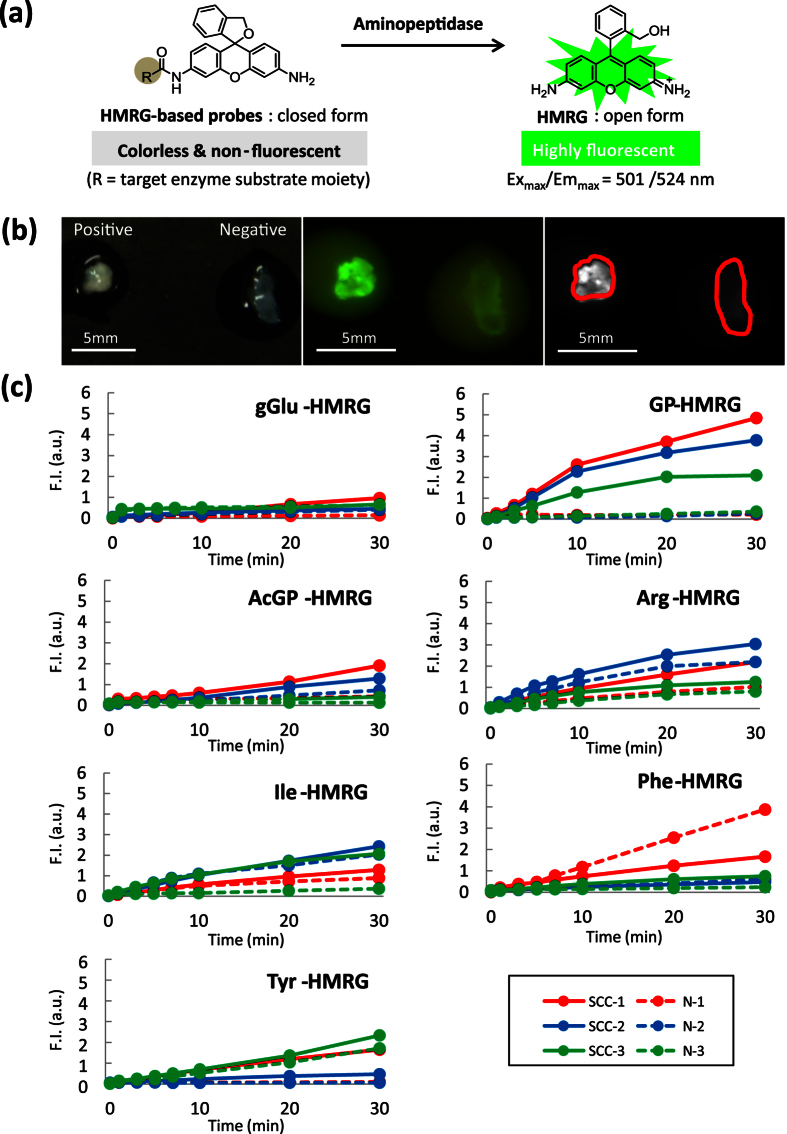
Screening of HMRG-based aminopeptidase-reactive fluorescent probes. (**a**) Activation of fluorescence of HMRG-based probes upon enzymatic reaction. (**b**) Fluorescence imaging of cancer-positive biopsy samples and cancer-negative biopsy samples. White-light image before spraying probe (left). Fluorescence images after spraying fluorescent probe under blue light (middle). Fluorescence images at 540 nm after spraying fluorescent probe; ROI are outlined in red (right). Tyr-HMRG was used as the fluorescent probe in these images. Scale bars, 5 mm. (**c**) Screening of aminopeptidase activity using human cancer-positive (SCC, solid line) and cancer-negative (N, dotted line) biopsy samples. Biopsy samples from the same patient are shown in the same color. F.I. (a.u.): fluorescence intensity (arbitrary units).

In order to find an aminopeptidase activity that is specific for ESCCs, and to ensure that the findings would be directly translatable to clinical application, we carried out screening using fresh biopsy samples taken from cancer-positive and negative sites during preoperative upper gastrointestinal endoscopic examination of patients. The biopsy samples were incubated with a series of HMRG-based activatable probes targeted to various candidate aminopeptidases. These probes exist in colorless, non-fluorescent spirocyclic forms, but are converted to a colored, highly fluorescent hydrolysis product HMRG, which emits green fluorescence, upon reaction with the targeted aminopeptidase (Fig. 1a). Since these biopsy samples were fresh, we expected that *in vivo* differences in enzymatic activities between cancer-positive and negative sites would be retained (Fig. 1b). The screening probes were targeted to γ-glutamyltranspeptidase (gGlu-HMRG), dipeptidylpeptidase IV (GP-HMRG), fibroblast activation protein (AcGP-HMRG), cathepsin H (Arg-HMRG), and other candidate peptidases (Ile-HMRG, Phe-HMRG, Tyr-HMRG). Among them, GP-HMRG showed a rapid, significant and specific fluorescence increase in tumor-positive samples, while the others did not (Fig. 1c).

**Figure 2:**
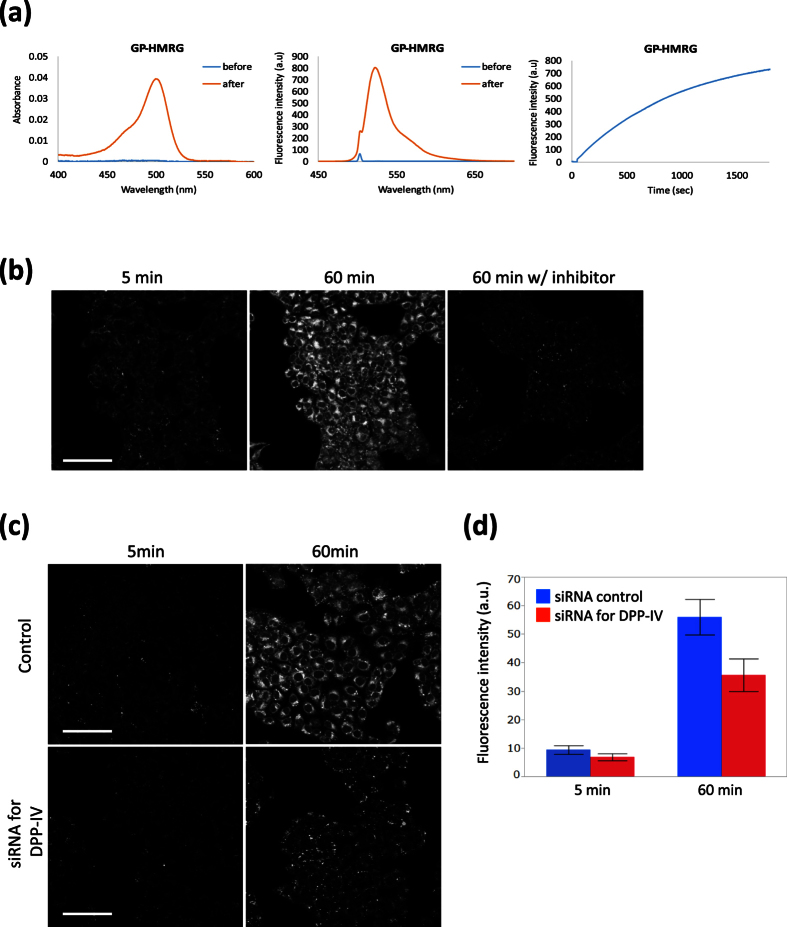
Fluorescence imaging of human esophageal squamous cell carcinoma cell line KYSE270 with GP-HMRG. (**a**) *In vitro* changes in absorption (left) and fluorescence (middle) spectra of GP-HMRG before and after addition of DPP-IV. (**b**) Confocal fluorescence images at 5 and 60 min after incubation with GP-HMRG (10 μM) in the absence and presence of inhibitor (100 μM). Drastic fluorescence activation of GP-HMRG was observed, and was significantly suppressed by the inhibitor. Scale bars, 100 μm. (**c**) Confocal fluorescence images of KYSE270 cells which had been pretransfected with siRNAs, followed by application of GP-HMRG (10 μM). Scale bars, 100 μm. (**d**) Change of fluorescence intensity in KYSE270 cells in (**b**). Data are mean fluorescence intensities (a.u.) ± SEM.

Next, we set out to confirm the target enzyme of GP-HMRG. Based on a previous report that dipeptide GP (GlyPro) is cleaved by DPP-IV^17^, we first confirmed that GP-HMRG can be activated by DPP-IV *in vitro* (Fig. 2a). Next, since there are only a few papers reporting up-regulation of DPP-IV in ESCCs^18^,^19^,^20^, we performed live-cell imaging of cultured esophageal cancer cells to confirm the reactivity of GP-HMRG and its specificity for DPP-IV. We observed fluorescence activation of GP-HMRG in human esophageal squamous cell carcinoma KYSE270 cells (Fig. 2b). In the presence of a DPP-IV inhibitor, this fluorescence activation was blocked. Similarly, we observed a significant decrease in the fluorescence signal in cells pre-transfected with DPP-IV siRNAs, compared to cells transfected with control siRNA (Fig. 2c,d). These results confirm that DPP-IV is responsible for the activation of GP-HMRG in fresh biopsy samples.

### Synthesis of a series of fluorescence probes for DPP-IV

The penultimate proline is indispensable for the substrate recognition by DPP-IV^17^, so we synthesized a series of candidate probes, EP-HMRG, KP-HMRG, YP-HMRG, LP-HMRG, and PP-HMRG, for further evaluation. We confirmed that all these probes exist in non-fluorescent spirocyclic form, but are converted to a highly fluorescent hydrolysis product, HMRG, upon reaction with DPP-IV (Supplementary Figs 1–4, Supplementary Table 1). Among these derivatives, EP-HMRG exhibited the lowest Michaelis constant (K_m_), showing the highest affinity for DPP-IV (Supplementary Table 2). Therefore, we selected EP-HMRG for further application.

### *Ex vivo* validation of DPP-IV-activatable probe EP-HMRG for detecting esophageal squamous cell carcinoma in biopsy samples

**Figure 3:**
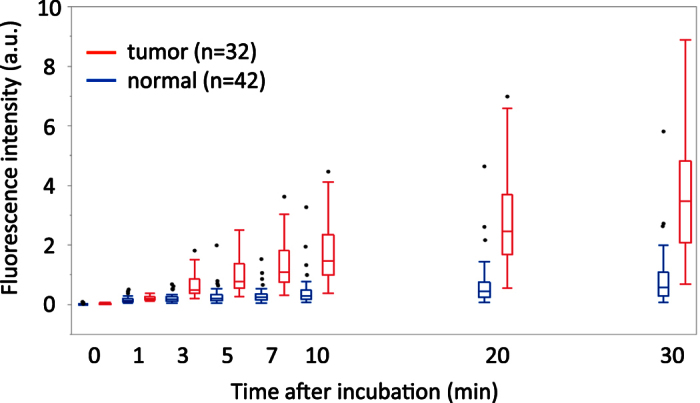
Fluorescence analysis of freshly resected biopsy samples of ESCCs from patients. Time-dependent changes in fluorescence intensity in biopsy samples after application of EP-HMRG (50 μM) (32 cancer-positive biopsy samples, red line; 42 cancer-negative biopsy samples, blue line). Mean fluorescence intensities (horizontal line within box), interquartile range (box) and range (error bars) are shown.

In order to validate EP-HMRG, we measured the fluorescence increase when it was applied to 74 biopsy samples, consisting of 32 samples diagnosed as positive for ESCCs and 42 negative samples. Significant fluorescence increases were observed with the cancer-positive samples, but not the cancer-negative samples, and the tumors were visualized with sufficient tumor-to-normal (T/N) fluorescence intensity ratios after 5 min (Fig. 3, Supplementary Table 3). Subsequent ROC analysis of the diagnostic performance of EP-HMRG for detecting ESCCs (Supplementary Fig. 5) gave sensitivity, specificity and accuracy values of 96.9%, 85.7% and 90.5%, respectively (Table 1). It is noteworthy that these values are comparable with or even better than those of conventional methods (Supplementary Table 4). These results clearly demonstrate the potential of EP-HMRG for clinical application.

### Fluorescence detection of human esophageal squamous cell carcinoma in resected specimens with EP-HMRG

**Figure 4:**
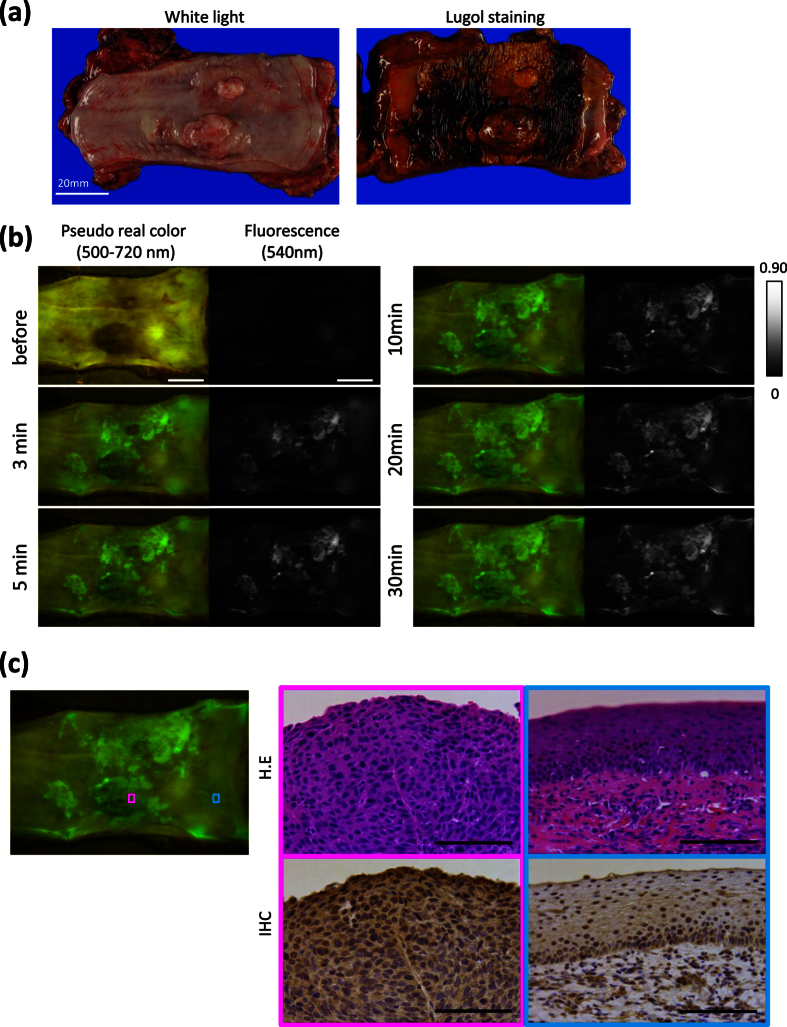
Fluorescence and histological analysis of freshly resected human ESCC specimen obtained at operation. (**a**) White-light image (WLI) and lugol dye staining: the pathological diagnosis was 0-I + IIc, 85 mm in size, pT1b. Scale bar, 20 mm. (**b**) Fluorescence images after spraying EP-HMRG (50 μM) under blue light: a rapid fluorescence increase was observed at the tumor lesion. Scale bar, 20 mm. (**c**) Histological analysis of boxed regions with strong fluorescence activation (pink box) or with no fluorescence activation (blue box). H&E (upper) and IHC staining for DPP-IV (bottom) revealed that the pink box region consisted of SCCs with strong DPP-IV expression (middle) and the blue box region was normal (right). Magnification ×400. Scale bar, 100 μm.

**Figure 5:**
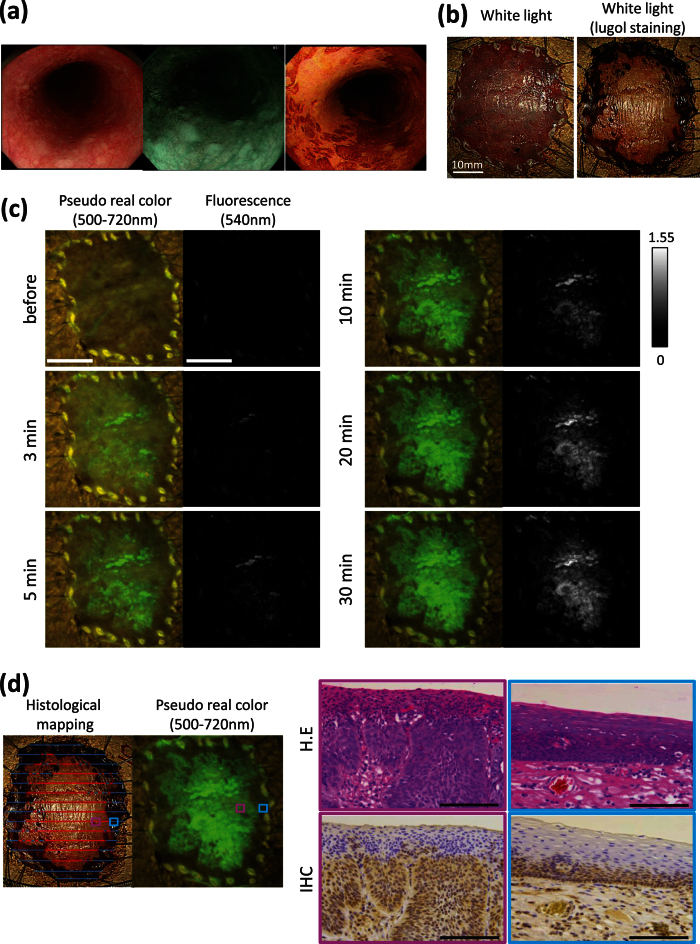
Fluorescence and histological analysis of freshly resected ESD specimen. (**a**) Endoscopic appearance. White light imaging (WLI) shows flat appearance and slight redness (left). Narrow band imaging (NBI) shows a brownish area (middle). Lugol dye shows a well-demarcated unstained area (right). (**b**) WLI and lugol staining: the pathological diagnosis was 0-IIb, 33 mm in size, pT1a. Scale bar, 10 mm. (**c**) Fluorescence images after spraying EP-HMRG (50 μM) under blue light: a rapid fluorescent increase was observed in the tumor lesion. Scale bar, 10 mm. (**d**) Red lines on histological mapping show SCCs and blue lines show no tumor (left). Histological analysis of boxed regions with strong fluorescence activation (pink box) or with no fluorescence activation (blue box). H&E (upper) and IHC staining for DPP-IV (bottom) revealed the pink box region was SCCs (middle) and the blue box region was normal (right). Magnification ×400. Scale bar, 100 μm.

We next investigated whether tumor-specific fluorescence imaging could be achieved simply by spraying EP-HMRG onto freshly resected specimens obtained from esophageal cancer patients during endoscopic submucosal dissection (ESD) or at surgical operation. A total of 44 specimens (30 surgical specimens and 14 ESD specimens) were obtained and examined (Figs 4 and 5).

Figure 4 shows an example of a specimen resected at operation: a 0-I + IIc lesion of 85 mm in size, located in the middle esophagus, was histologically diagnosed as well-differentiated squamous cell carcinoma (SCC), pT1b. After spraying of EP-HMRG, the tumor could be clearly detected within a few minutes. The pattern of the observed fluorescence signal closely matched that of lugol voiding lesions (Fig. 4a,b). We also immunohistochemically confirmed expression of DPP-IV in the SCC (Fig. 4c). Pathological findings were also consistent with the real-time fluorescence detection of the tumor by fluorescence endoscopy (Supplementary Video 1). Further, co-incubation with a DPP-IV inhibitor (DPP-IV Inhibitor IV) blocked the fluorescence increase in another resected specimen obtained at operation (Supplementary Fig. 6: a 0-IIc lesion 30 mm in size, located in the middle esophagus, was histologically diagnosed as SCC, pT1a). These results demonstrated that EP-HMRG is cleaved by DPP-IV expressed in the tumor in resected specimens, thereby generating a fluorescence signal.

Figure 5 shows an example of early ESCCs removed by ESD: a 0-IIb lesion 33 mm in size, located in the middle esophagus, which was histologically diagnosed as SCC, pT1a. Since this lesion was flat and slightly red, it was difficult to detect by white light imaging (WLI), but was highlighted in NBI or by lugol staining (Fig. 5a,b). Just 5 min after spraying EP-HMRG onto this resected specimen, the tumor lesion was clearly and specifically visualized (Fig. 5c). Indeed, the fluorescence signal was sufficiently strong to be seen with the naked eye. The fluorescence-positive site matched well with the lugol voiding lesions and with the pathologically identified cancer-positive site (Fig. 5d). Furthermore, the SCC showed strong immunostaining of DPP-IV.

## Discussion

In this report, we adopted a screening strategy to find ESCC-specific enzymatic activities, using freshly resected specimens from patients and a series of activatable aminopeptidase-targeting fluorescence probes, and we identified DPP-IV, a serine protease that cleaves penultimate L-proline at the N-terminus of polypeptides^21^), as a suitable candidate. It has been reported that DPP-IV is overexpressed in several kinds of cancer tissue, including prostate carcinomas, thyroid carcinomas, and ESCCs^18^,^19^,^20^,^22^,^23^, but the present work is the first to show that DPP-IV can be utilized for visualizing esophageal cancer.

Detecting ESCCs at an early stage is critical for preventing development of advanced cancer and for enabling minimally invasive, curative treatment^2^,^3^,^4^,^5^. Several techniques have been established to detect early-stage ESCCs through endoscopic screening, but they have various limitations. Lugol chromoendoscopy has sensitivity, specificity, and diagnostic accuracy values of 94.2%, 64.0% and 68.0%, respectively (Supplementary Table 4). However, discomfort (esophageal burning sensation) has frequently been reported after lugol chromoendoscopy, and allergic reaction to iodine can sometimes occur^8^,^9^,^10^. Further, instillation of lugol dye close to the upper esophagus must be avoided due to the risk of bronchospasm or aspiration, and accurate diagnosis is difficult because of the wide variation in staining patterns^24^,^25^, which might lead to unnecessary biopsies. On the other hand, NBI offers non-invasive observation with sensitivity, specificity, and diagnostic accuracy values of 88.3%, 75.2% and 77.0%, respectively. However, especially in the case of magnifying NBI, these values may vary substantially depending upon the skill and experience of endoscopists (Supplementary Table 4). Our simple and convenient method using an activatable fluorescence probe, EP-HMRG, provided sensitivity, specificity and accuracy values of 96.9%, 85.7% and 90.5% at just 5 min after topical spraying of the probe. These values are comparable to or even better than those of lugol chromoendoscopy and NBI.

Several successful applications of fluorescent probes for *in vivo* cancer imaging in patients have been reported^13^,^14^. However, in all cases, the probes have to be intravenously injected, so that thorough safety studies are mandatory before pilot studies in humans can be carried out. Also, relatively large amounts of the probes (0.1–0.3 mg/kg) were used, compared to our topically sprayed probe (0.5 mg/one patient), and quite long detection periods were required after probe administration (2–8 hrs). These factors have made it difficult to accumulate sufficient numbers of patients to properly evaluate the sensitivity and specificity of these novel techniques in humans. In contrast, the topical application of our probe at a substantially lower dose and its rapid reaction would be favorable for routine clinical application during endoscopic examination, including screening checks for ESCCs. The rapid and dramatic fluorescence activation would enable easy incorporation of this technique into surgical or endoscopic resection procedures, e.g., for evaluation of the surgical margin during endoscopic submucosal dissection (ESD) and surgical operation.

A unique feature of our strategy is that the selected imaging probe is directly translatable to clinical application. Previous approaches to identify cancer-related biomarkers, such as a specific receptor, protein or metabolic product, by means of biochemical and omics analysis of cancer model cells, animal models, or patient’s sample, often require prolonged research and may nevertheless be unsuccessful. In contrast, our strategy of using activatable, aminopeptidase-targeted fluorescence probes to visualize cancers in fresh human specimens is directly translatable to the clinical context, because we can observe and compare a specific enzymatic activity at cancer sites as compared to normal sites in the same sample in detail. This provides basic information that would be helpful in designing individualized treatment regimens to improve the outcome for patients.

The fluorescence signal obtained with EP-HMRG corresponded well with pathologically cancer-positive lesions, but we have noticed that the fluorescence signal is not uniform within cancer-positive lesions. One possible reason for this would be differences of histological type. According to Goscinski *et al*.^18^,^19^, and Augoff *et al*.^20^, the expression level of DPP-IV varies depending on the differentiation status of ESCC cell lines. Other possible reasons would be differences of grade of malignancy or depth of tumor invasion. In immunohistochemistry, strong immunostaining of DPP**-**IV was observed in SCC, whereas DPP-IV expression was confined to the basal and parabasal layers in normal regions. Based on this, we speculate that the difference in DPP-IV localization between ESCCs and normal esophageal epithelium contributes to the high T/N ratio; DPP-IV in the normal epithelium tends to be sequestered to basal cells that are not easily accessed by the probe, while DPP-IV in ESCCs tends to be over-expressed or exposed near or at the surface of the epithelium, so that the probe can be easily hydrolyzed to the fluorescent product. Therefore, it will be of interest to investigate the relationship between fluorescence intensity and histological type, grade of malignancy or depth of tumor invasion in future studies.

To our knowledge, this is the first report to describe the strategy of screening activatable probes with fresh human clinical specimens to find suitable molecular targets for cancer-specific fluorescence imaging. Our newly developed probe for DPP-IV, EP-HMRG, is a good candidate for clinical application, not only for the diagnosis of early ESCCs under the endoscope, but also for evaluation of the surgical margin during ESD and surgical operation. Further studies to evaluate the safety and biokinetics of this probe are in progress.

## Methods

### Patients and lesions

This study was conducted with the approval of the Research Ethics Committee of the University of Tokyo and registered in the UMIN Clinical Trials Registry (registration number: UMIN000012645; http://www.umin.ac.jp/ctr/index.htm). All experiments were performed in accordance with guidelines and regulations approved by the Research Ethics Committee of the University of Tokyo. Informed consent was obtained from all patients. Esophageal cancer patients examined or treated at the University of Tokyo Hospital in Tokyo, Japan, were prospectively included in this study. Patients with ESCCs were included, but patients with esophageal adenocarcinoma or esophago-gastric junctional cancer were excluded.

### Materials

General chemicals were of the best grade available, supplied by Tokyo Chemical Industries, Wako Pure Chemical or Aldrich Chemical Company, and were used without further purification. Dimethyl sulfoxide (DMSO, fluorometric grade) used for the spectrometric measurements and for preparing stock solutions was purchased from Dojindo. DPP**-**IV was purchased from Sigma-Aldrich Japan K.K. (D4943: Tokyo, Japan), and K579 (DPP-IV inhibitor) was purchased from CalbioChem.

### Instruments

^1^H NMR spectra were recorded on a JNM-LA300 (JEOL) instrument (300 MHz for ^1^H NMR) or JNM-LA400 instrument (400 MHz for ^1^H NMR). Mass spectra (MS, ESI-TOF) were measured with a JMS-T100LC AccuTOF (JEOL). Absorption spectra were obtained with a UV-1650PC UV/Vis spectrometer (Shimadzu), and fluorescence spectra were obtained with a F4500 fluorescence spectrometer (Hitachi). LC-MS analysis were performed on a reverse-phase column (Inertsil C18, GL Sciences (Tokyo, Japan)), fitted on an Agilent Technologies 1200 series/6130 Quadrupole (LC/MS) system, using a linear gradient of eluent A (0.1% formic acid in H_2_O) and eluent B (0.1% formic acid in 80% acetonitrile, 20% H_2_O) (A/B: 95/5 to 5/95 in 17.5 min). Detected at 490 nm. All experiments were carried out at 298 K, unless otherwise specified.

### Cell lines and culture

The established cell line KYSE270, originated from well-differentiated human esophageal squamous cell carcinoma, was provided by Y. Shimada, Kyoto University, Japan^26^. KYSE270 cells were grown in a 1-to-1 mixture of Ham’s F12 medium and RPMI1640 medium containing 2% fetal bovine serum (FBS), penicillin (100 U/ml) and streptomycin (100 μg/ml) at 37 °C in an atmosphere of 5% CO_2_ in air.

### RNA interference

KYSE270 cells were seeded on 8-well μ-slides (ibidi) and transfected with 10 nM DPP-IV-targeted siRNA (CD26 siRNA (h), Santa Cruz, SC-42762) or control siRNA (BannoNegacon, RNAi Inc.) using Lipofectamine RNAiMAX transfection reagent (Invitrogen, #13778030). Thirty hours after transfection, the cells were used for fluorescence imaging.

### Confocal live cell imaging

Cells were seeded on a glass-bottomed dish and cultured for 2 days, then incubated with 10 μM GP-HMRG in RPMI1640 containing 0.1% v/v DMSO as a co-solvent (when necessary, cells were co-incubated with 100 μM DPP-IV inhibitor). Fluorescence images were captured at 5 or 60 min after application of the probe with a Leica Application Suite Advanced Fluorescence (LAS-AF) microscope with a TCS SP5 unit and an oil immersion objective lens (×40, numerical aperture 1.25, Leica). The excitation and emission wavelengths were 488 nm and 500–580 nm, respectively. The light source was a white-light laser.

### Fluorescence imaging with biopsy samples

Biopsy samples from patients were freshly taken from both cancer-positive sites and negative sites during preoperative upper endoscopic examination. A 50 μM probe solution (50 μl) in PBS containing 0.5% v/v DMSO as a co-solvent was dropped onto each sample, and fluorescence images were obtained before and at 1, 3, 5, 7, 10, 20 and 30 min after application of the probe with the Maestro *In Vivo* Imaging System (PerkinElmer, Massachusetts, USA). The blue-filter setting (excitation: 435 to 480 nm; emission: 490 nm long-pass) was used. The tunable filter was automatically stepped in 10-nm increments, from 500 to 720 nm, while the camera sequentially captured images at each wavelength interval. The diagnostic performance of EP-HMRG was evaluated by ROC analysis of average fluorescence intensity of the biopsy samples at 540 nm (Fig. 1b).

### Fluorescence imaging with resected specimens obtained by ESD or at surgical operation

Esophageal specimens from esophageal cancer patients were resected during ESD or surgical operation. A 50 μM solution of EP-HMRG (1000 μl) in PBS containing 0.5% v/v DMSO as a co-solvent was sprayed onto the freshly resected specimens, and fluorescence images were obtained before and at 3, 5, 10, 20 and 30 min after spraying, as described above. A mixture of EP-HMRG (50 μM) and DPP-IV inhibitor (50 μM, Calbiochem) was used to examine specificity of EP-HMRG for DPP-IV on the specimens.

### Real-time fluorescence endoscopy

A fluorescence endoscopic system (Olympus Corp.) equipped with an in-house-developed fluorescence detection system was used for this study. Excitation and emission filters were 450 to 490 nm and 520 to 600 nm, respectively. Fluorescence images were captured with the endoscope at 10 min after spraying EP-HMRG.

### Histological analysis

Resected specimens were evaluated pathologically with hematoxylin and eosin (H&E) staining to confirm the existence of cancer. These specimens were fixed in 10% neutral buffered formalin, embedded in paraffin and sliced at 4 μm thickness. The tissue sections were deparaffinized, and stained with hematoxylin and eosin for histopathologic evaluation. Immunohistochemistry (IHC) of DPP-IV was performed for comparison with the pathological diagnosis. The distribution of carcinoma evaluated pathologically in the resected specimen was also compared to that of fluorescence-positive and lugol-voiding regions.

### Immunohistochemistry

After tissue sections had been prepared as described above, the slides were immersed in immunosaver buffer and microwaved at 98 °C for 20 minutes for antigen retrieval. The VECTASTAIN Elite ABC (Avidin Biotinylated enzyme Complex) system was used for staining tissue according to the manufacturer’s instructions. Briefly, the slides were incubated in 3% H_2_O_2_/H_2_O for 30 min to quench endogenous peroxidase activity, and an Avidin/Biotin blocking system was used to eliminate non-specific staining. The sections were incubated with primary antibody against CD26 (DPP-IV) (rabbit polyclonal antibody, H-270 (Santa Cruz, CA)) at 1:500 dilution for 2 hours at room temperature. Sample without primary antibody was also prepared as a negative control. Normal small intestine, known to be DPP-IV-positive, was used as a positive control. 3,3′-Diaminobenzidine (DAB) was used as a chromogen and hematoxylin was applied for counterstainng.

### Statistical analysis

Continuous data were compared using the Wilcoxon rank-sum test. Receiver operating characteristic (ROC) curves was used to determine a cutoff value. Sensitivity, specificity and accuracy were calculated by using ROC analysis. A value of P < 0.050 (2-sided) was regarded as statistically significant. All analyses were performed using JMP pro software version 11 (SAS Institute, Cary, North Carolina, USA).

## Additional Information

**How to cite this article**: Onoyama, H. *et al*. Rapid and sensitive detection of early esophageal squamous cell carcinoma with fluorescence probe targeting dipeptidylpeptidase IV. *Sci. Rep.* **6**, 26399; doi: 10.1038/srep26399 (2016).

## Supplementary Material

Supplementary Information

Supplementary Video 1

## Figures and Tables

**Table 1:** Diagnostic performance of EP-HMRG for detection of esophageal cancer.

	5 min		10 min		30 min	
AUC	0.93		0.93		0.95	
Cut off	0.37		0.67		1.77	
Sensitivity	96.9%	(31/32)	96.9%	(31/32)	96.9%	(31/32)
Specificity	85.7%	(36/42)	83.3%	(35/42)	90.5%	(38/42)
Accuracy	90.5%	(67/74)	89.2%	(66/74)	93.2%	(69/74)
PPV^*^	83.8%	(31/37)	81.6%	(31/38)	88.6%	(31/35)
NPV^**^	97.3%	(36/37)	97.2%	(35/36)	97.4%	(38/39)

The values are positive rate. *PPV, positive predictive value. **NPV, negative predictive value.
