# Supplementary material for: Rapid and sensitive detection of early esophageal squamous cell carcinoma with fluorescence probe targeting dipeptidylpeptidase IV
Source: Sci Rep. 2016 Jun 1;6:26399. doi: 10.1038/srep26399 (PMC4887889; doi:10.1038/srep26399)
Supplement: Supplementary Information [file srep26399-s1.doc]

**Supplementary Materials for**

Rapid and sensitive detection of esophageal cancer with fluorescence probes targeting dipeptidylpeptidase IV

**Authors:** Haruna Onoyama, Mako Kamiya, Yugo Kuriki, Toru Komatsu, Hiroyuki Abe, Yosuke Tsuji, Koichi Yagi, Yukinori Yamagata, Susumu Aikou, Masato Nishida, Kazuhiko Mori, Hiroharu Yamashita, Mitsuhiro Fujishiro, Sachiyo Nomura, Nobuyuki Shimizu, Masashi Fukayama, Kazuhiko Koike, Yasuteru Urano^*^, Yasuyuki Seto^*^

*To whom correspondence should be addressed. E-mail: uranokun@m.u-tokyo.ac.jp (Y.U.); seto-tky@umin.ac.jp (Y.S.)

**This file includes:**

**Supplementary Figure 1.** pH dependency of absorption and emission spectra of XP-HMRG.

**Supplementary Figure 2.** pH dependency of absorbance at 496 nm of XP-HMRG.

**Supplementary Figure 3.** Changes in absorption and fluorescence spectra of XP-HMRG before and after addition of DPP-IV.

**Supplementary Figure 4.** LC-MS analysis of reaction mixture of XP-HMRG with DPP-IV.

**Supplementary Figure 5.** ROC curve of EP-HMRG observation for detection of esophageal cancer.

**Supplementary Figure 6.** Examination with DPP-IV inhibitor using fresh human resected specimen of ESCCs obtained at operation.

**Supplementary Table 1.** Photochemical properties of XP-HMRG.

**Supplementary Table 2.** Comparison of kinetic parameters of DPP-IV probes.

**Supplementary Table 3.** Mean fluorescence intensity of biopsy samples.

**Supplementary Table 4.** Observation methods used for esophageal cancer detection by upper endoscopy.

**Supplementary Video 1** **(.avi format).** Dynamic fluorescence endoscopy of freshly resected specimen of human ESCC at 10 min after spraying EP-HMRG.

**SUPPLEMENTARY METHODS**

**Synthesis and characterization of aminopeptidase activatable probes**

DPP-IV substrates were synthesized according to the literature (*S1*).

**GP-HMRG.** ^1^H NMR (400 MHz, CD_3_OD): δ 2.07-2.21 (m, 3H), 2.33-2.37 (m, 1H), 3.56-3.74 (m, 2H), 3.96 (s, 2H), 4.36 (s, 2H), 4.65-4.67 (m, 1H), 6.92 (s, 1H), 7.05 (d, 1H, *J* = 8.8 Hz), 7.34 (t, 3H, *J* = 9.0 Hz), 7.52 (s, 1H), 7.59-7.60 (m, 1H), 7.70-7.72 (m, 2H), 8.39 (s, 1H). HRMS (ESI^+^): calcd for [M+H]^+^, 471.20323 ; found, 471.20193 (-1.30 mmu).

**EP-HMRG.** ^1^H NMR (400 MHz, CD_3_OD): δ 2.01-2.29 (m, 5H), 2.40-2.41 (m, 1H), 2.66 (t, 2H, *J* = 7.1 Hz), 3.79 (m, 2H), 4.36 (s, 2H), 4.43 (t, 1H, *J* = 5.9 Hz), 4.67-4.68 (m, 1H), 6.92 (s, 1H), 7.02 (d, 1H, *J* = 9.3 Hz), 7.34 (t, 3H, *J* = 9.7 Hz), 7.51 (s, 1H), 7.57-7.61 (m, 1H), 7.71-7.77 (m, 2H), 8.42 (s, 1H). HRMS (ESI^+^): calcd for [M+H]^+^, 543.22436 ; found, 543.21944 (-4.92 mmu).

**KP-HMRG.** ^1^H NMR (400 MHz, CD_3_OD): δ 1.62-1.67 (m, 2H), 1.70-1.78 (m, 2H), 1.96-1.98 (m, 2H), 2.07-2.10 (m, 2H), 2.14-2.21 (m, 1H), 2.40 (br s, 1H), 3.00 (t, 2H, *J* = 7.6 Hz), 3.70-3.72 (m, 1H), 3.81-3.84 (m, 1H), 4.33 (t, 3H, *J* = 7.1 Hz), 4.67 (br s, 1H), 6.95 (s, 1H), 7.05 (d, 1H, *J* = 9.3 Hz), 7.36 (t, 3H, *J* = 8.5 Hz), 7.57-7.58 (m, 2H), 7.70-7.73 (m, 2H), 8.36 (s, 1H). HRMS (ESI^+^): calcd for [M+H]^+^, 542.27673 ; found, 542.28086 (+4.14 mmu).

**YP-HMRG.** ^1^H NMR (400 MHz, CD_3_OD): δ 1.93-2.14 (m, 3H), 2.29-2.30 (m, 1H), 2.96-2.99 (m, 1H), 3.18-3.21 (m, 2H), 3.63-3.68 (m, 1H), 4.37 (t, 3H, *J* = 6.3 Hz), 4.65 (br s, 1H), 6.77-6.78 (m, 3H), 6.97 (s, 1H), 7.05 (d, 1H, *J* = 10.2 Hz), 7.21 (d, 2H, *J* = 8.3 Hz), 7.35-7.38 (m, 3H), 7.53 (d, 1H, *J* = 8.3 Hz), 7.59 (t, 1H, *J* = 6.6 Hz),7.71-7.73 (m, 2H), 8.49 (s, 1H). HRMS (ESI^+^): calcd for [M+H]^+^, 577.24509 ; found, 577.24834 (+3.24 mmu).

**LP-HMRG.** ^1^H NMR (400 MHz, CD_3_OD): δ 1.07 (d, 6H, *J* = 4.9 Hz), 1.69-1.89 (m, 3H), 2.05-2.11 (m, 2H), 2.16-2.24 (m, 1H), 2.37-2.42 (m, 1H), 3.63-3.69 (m, 1H), 3.80-3.83 (m, 1H), 4.27 (dd, 1H, *J* = 3.9, 9.3 Hz), 4.35 (s, 2H), 4.66-4.68 (m, 1H), 6.95 (s, 1H), 7.04 (d, 1H, *J* = 9.3 Hz), 7.34-7.36 (m, 3H), 7.51 (d, 1H, *J* = 8.8 Hz), 7.58 (t, 1H, *J* = 6.9 Hz), 7.67-7.75 (m, 2H), 8.49 (s, 1H). HRMS (ESI^+^): calcd for [M+H]^+^, 527.26583 ; found, 527.26964 (+3.81 mmu).

**PP-HMRG.** ^1^H NMR (400 MHz, CD_3_OD): δ 1.97-2.23 (m, 8H), 2.38-2.43 (m, 1H), 2.53-2.58 (m, 1H), 3.62-3.70 (m, 1H), 3.72-3.77 (m, 1H), 4.35 (s, 2H), 4.62-4.66 (m, 2H), 6.94 (s, 1H), 7.04 (d, 1H, *J* = 9.3 Hz), 7.34-7.36 (m, 3H), 7.51 (d, 1H, *J* = 8.3 Hz), 7.58 (t, 1H, *J* = 6.6 Hz), 7.68-7.75 (m, 2H), 8.42 (s, 1H). HRMS (ESI^+^): calcd for [M+H]^+^, 511.23453 ; found, 511.23291 (-1.62 mmu).

**Y-HMRG.** ^1^H NMR (300 MHz, CD_3_OD+KOD): δ 2.71 (dd, 1H, *J =* 3.3, 6.6 Hz), 2.90 (dd, 1H, *J =* 3.3, 6.6 Hz), 3.58 (t, 1H, *J =* 6.6 Hz), 5.24 (s, 2H), 6.42 (dd, 1H, *J =* 2.2, 8.8 Hz), 6.51 (d, 1H, *J =* 2.2 Hz), 6.56 (d, 2H, *J* = 8.1 Hz), 6.64 (d, 1H, *J* = 8.8 Hz), 6.81 (d, 2H, *J* = 8.8 Hz), 6.87 (d, 2H, *J* = 8.1 Hz), 7.03 (ddd, 1H, *J* = 2.0, 8.3, 14.1 Hz), 7.28 (t, 1H, *J =* 6.2 Hz), 7.38-7.41 (m, 2H), 7.56 (dd, 1H, J = 2.2, 12.5 Hz). HRMS (ESI^+^): calcd for [M+H]^+^, 480.19233 ; found, 480.19658 (+4.24 mmu).

**R-HMRG.** ^1^H NMR (300 MHz, CD_3_OD+KOD): δ 1.71-1.80 (m, 4H), 3.14 (t, 2H, *J =* 6.2 Hz), 3.47 (t, 1H, *J =* 5.9 Hz), 5.25 (s, 2H), 6.42 (dd, 1H, *J =* 2.2, 8.8 Hz), 6.51 (d, 1H, *J =* 2.2 Hz), 6.65 (d, 1H, *J* = 2.2 Hz), 6.65 (d, 1H, *J* = 8.1 Hz), 6.80-6.84 (m, 2H), 7.11 (td, 1H, *J* = 5.3, 2.7 Hz), 7.28 (t, 1H, *J =* 7.3 Hz), 7.36-7.44 (m, 2H), 7.62 (t, 1H, J = 2.2 Hz). HRMS (ESI^+^): calcd for [M+H]^+^, 473.23011 ; found, 473.23222 (+2.11 mmu)

**Kinetic Assay (Supplementary Table 2).** Probe (XP-HMRG) was dissolved at various concentrations in 3 mL total volume of 10 mM Tris buffer (pH 7.4), containing 1% DMSO as a cosolvent. 3 µL of DPP-IV (SIGMA D4943) was added to the solution, and the initial velocity was calculated from the changes in fluorescence intensity (Ex / Em = 501 nm / 524 nm). The obtained values were plotted against probe concentration, and fitted to a Michaelis Menten curve. The kinetic parameters were calculated by use of the Michaelis−Menten equation:

V = Vmax *[S]/(Km + [S])

where V is initial velocity, and [S] is substrate concentration

**SUPPLEMENTARY FIGURES**

**(e)**

**(f)**

**Supplementary Figure 1.** pH dependency of absorption and emission spectra of XP-HMRG. (a) GP-HMRG, (b) EP-HMRG, (c) KP-HMRG, (d) YP-HMRG, (e) LP-HMRG, (f) PP-HMRG. These spectra were measured at various pH values in 0.1 M sodium phosphate buffer, containing <0.1% DMSO as a cosolvent. Excitation wavelength was 496 nm.

**Supplementary Figure 2.** pH dependency of absorbance at 496 nm of XP-HMRG. (a) GP-HMRG, (b) EP-HMRG, (c) KP-HMRG, (d) YP-HMRG, (e) LP-HMRG, (f) PP-HMRG.

**(c)**

**(b)**

**(d)**

**(e)**

**(a)**

**Supplementary Figure 3.** Changes in absorption (left) and fluorescence (middle) spectra of XP-HMRG before and after addition of DPP-IV. To a 1 μM solution of EP-HMRG (a), KP-HMRG (b), YP-HMRG (c), LP-HMRG (d), PP-HMRG (e) in 3 mL of PBS(-), pH 7.4, containing 0.1% DMSO as a cosolvent, 5 µL of DPP IV (SIGMA D4943) was added. (Right)　Time course of fluorescence intensity upon addition of DPP-IV was monitored at excitation/emission wavelengths of 501 nm /524 nm, respectively. Arrow indicates the time of DPP-IV addition.

**(b)**

**(a)**

**(d)**

**(e)**

**(f)**

**(c)**

**Supplementary Figure 4.** LC-MS analysis of reaction mixture of XP-HMRG with DPP-IV. (a) GP-HMRG, (b) EP-HMRG, (c) KP-HMRG, (d) YP-HMRG, (e) LP-HMRG, (f) PP-HMRG. To a　10 µM probe solution in 20 µL PBS,　1 µL of DPP IV (SIGMA D4943) was added. The reaction mixture was incubated at 37 ºC for 150 min, and then the enzymatic reaction was stopped by adding 10% formic acid in MeOH. LC-MS analysis was performed with a linear gradient of eluent A (0.1% formic acid in H_2_O) and eluent B (0.1% formic acid in 80% acetonitrile, 20% H2O); A/B = 95/5 to 5/95 in 17.5 min. Detected at 490 nm. The chromatogram demonstrates the enzymatic conversion of XP-HMRG to HMRG.

30min

5 min

10 min

30 min

**Supplementary Figure 5.** ROC curve of EP-HMRG observation for detection of esophageal cancer.

a

**b**

**c**

0

**Supplementary Figure 6.** Examination of freshly resected human ESCC specimen (obtained at operation) in the presence of DPP-IV inhibitor. (a) WLI and Lugol dye imaging of resected specimen. (b) Inhibition method (c) Fluorescence images after spraying EP-HMRG or inhibitor under blue light; the lower half of the specimen shows high brightness and the upper half is non-fluorescent. Scale bar, 20 mm

**SUPPLEMENTARY TABLE**

**Supplementary Table 1.** Photochemical properties of XP-HMRG.

|  | Absorption  Maximum  (nm) | Emission  Maximum  (nm) | Fluorescence  Quantum  Yield^b)^ | p*K*_cycl_ |
| --- | --- | --- | --- | --- |
| GP-HMRG^a)^ | 496 | 528 | 0.31 | 5.0 |
| EP-HMRG ^a)^ | 496 | 528 | 0.32 | 5.1 |
| KP-HMRG ^a)^ | 496 | 529 | 0.32 | 4.9 |
| YP-HMRG ^a)^ | 496 | 529 | 0.22 | 5.0 |
| LP-HMRG ^a)^ | 496 | 529 | 0.33 | 4.9 |
| PP-HMRG ^a)^ | 496 | 529 | 0.32 | 5.0 |
| HMRG^(2^*^7^*) | 501 | 524 | 0.81 | 8.1 |

a) Measured in 0.1 M sodium phosphate buffer, pH 2.0, containing 1% DMSO as a cosolvent. b) Relative fluorescence quantum yield determined by using fluorescein (Φ_fl_ = 0.85) as a reference.

**Supplementary Table 2.** Comparison of kinetic parameters of DPP-IV probes.

|  | K_m_ (μM) ^a)^ |
| --- | --- |
| GP-HMRG | 29.6 |
| EP-HMRG | 17.7 |
| KP-HMRG | 38.0 |
| YP-HMRG | 27.3 |
| LP-HMRG | 28.6 |
| PP-HMRG | 20.5 |

a) Measured in 10 mM Tris buffer (pH 7.4), containing 1% DMSO as a cosolvent.

**Supplementary Table 3.** Mean fluorescence intensity of biopsy samples.

|  | Mean FI of  positive biopsy | Mean FI of  negative biopsy | Wilcoxon rank-sum test |
| --- | --- | --- | --- |
| Before | 0.04 | 0.02 | P<0.0001 |
| 1min | 0.23 | 0.18 | P=0.0015 |
| 3min | 0.62 | 0.24 | P<0.0001 |
| 5min | 0.97 | 0.31 | P<0.0001 |
| 7min | 1.28 | 0.34 | P<0.0001 |
| 10min | 1.76 | 0.48 | P<0.0001 |
| 20min | 2.93 | 0.72 | P<0.0001 |
| 30min | 3.84 | 0.91 | P<0.0001 |

**Supplementary Table 4.** Observation methods used to detect esophageal cancer by upper endoscopy.

|  | Sensitivity | Specificity | Accuracy |
| --- | --- | --- | --- |
| Conventional endoscopy  (WLI) | 55.2%^[6]^ | 63.2%^[6]^ | 56.5%^[6]^  30～50%^[12]^ |
| Lugol' staining | 94.2%^[7]^ | 64.0%^[7]^ | 68.0%^[7]^ |
| Non-magnifying  NBI | 88.3%^[7]^ | 75.2%^[7]^ | 77.0%^[7]^ |
| Magnifying  NBI | 97.2%^[6]^  Exp:100%^[12]^  Less :69.2%^[12]^ | 42.1%^[6]^  Exp:85.3%^[12]^  Less:93.9%^[12]^ | 88.9%^[6]^  Exp:87.0%^[12]^  Less :92.0%^[12]^ |
| EP-HMRG* | 96.9% | 85.4% | 90.5% |

* Values after 5min incubation.

**Exp: experienced endoscopists, Less: less experienced endoscopists

Data are from Muto et al. (*6*), Nagami et al. (*7*), and Ishihara et al. (*12*).

**Supporting Reference**

**S1.** Sakabe, M. et al. Rational design of highly sensitive fluorescence probes for protease and glycosidase based on precisely controlled spirocyclization. *J. Am. Chem. Soc.* **135**, 409–414 (2012).
